# Harmine Hydrochloride from *Peganum harmala* L. Targets NFAT1 for Proteasomal Degradation to Suppress Ovarian Cancer Progression

**DOI:** 10.3390/ph19091341

**Published:** 2026-08-24

**Authors:** Xingli Zhang, Huili Zhu, Chengshu Yu, Shuang Huang, Dongdong Fang

**Affiliations:** 1 Institute of Interdisciplinary Integrative Medicine Research, Shanghai University of Traditional Chinese Medicine, Shanghai 201203, China; 2 Department of Anatomy and Cell Biology, University of Florida College of Medicine, Gainesville, FL 32610, USA

**Keywords:** harmine hydrochloride, NFAT1, ovarian cancer, apoptosis, migration

## Abstract

**Background/Objectives**: Ovarian cancer is a prevalent gynecological malignancy with high mortality rates worldwide. Harmine hydrochloride (HMH), a tricyclic β-carboline alkaloid derived from the seeds of the medicinal plant *Peganum harmala* L., has demonstrated anti-tumor activity. This study investigated the effect of HMH on ovarian cancer and elucidated its underlying molecular mechanism. **Methods**: The anti-ovarian cancer effects of HMH were evaluated using the CCK-8 assay, cell cycle and apoptosis analyses, as well as a cell migration assay. The RNA-seq was utilized to explore the mechanisms of HMH. Western blot, qRT-PCR and IF were employed for further validation of the expression levels of relevant molecules. The Kaplan–Meier (KM) curve was conducted to evaluate the possible link between NFAT1 levels and the prognosis of ovarian cancer. The biological functions of NFAT1 were explored through siRNA knockdown experiments. Auto-dock molecular docking and DARTS were used to analyze the direct interaction between HMH and NFAT1. The in vivo efficacy of HMH was examined through bioluminescence imaging of intraperitoneal xenografts established with luciferase-expressing HEY cells. **Results**: HMH induced apoptosis, arrested the cell cycle, and suppressed migration of HEY and CAOV3 cells. NFAT1 was highly expressed in the nucleus of HEY and CAOV3 cells and promoted ovarian cancer progression. In addition, low NFAT1 expression levels had significantly improved overall survival and progression-free survival. Moreover, HMH reduced NFAT1 expression and reversed Ion-induced NFAT1 levels in ovarian cancer cell nuclei. The molecular docking and DARTS results suggested that HMH binds to NFAT1. Treatment with MG132 effectively blocked HMH-induced NFAT1 downregulation. Furthermore, HMH effectively blocked intraperitoneal xenograft development, and extended the life span of animals bearing HEY/Luc cells, when used alone or in combination with CDDP. **Conclusions**: The research indicated that HMH exerts anti-ovarian cancer effects, at least in part, by targeting NFAT1. Additionally, our research revealed that NFAT1 promotes ovarian cancer progression, and plays a significant role in HMH’s anti-cancer activity. These results may aid in the development of novel candidates for ovarian cancer and identify a new target for ovarian cancer treatment.

## 1. Introduction

Ovarian cancer remains the most lethal gynecological malignancy [1]. According to reports, over 324,398 patients are diagnosed with ovarian cancer in 2022, and more than 206,839 patients die from it [2]. Due to the absence of significant clinical symptoms and effective screening indicators in the early stages, approximately 75% of patients are diagnosed in advanced stages [3]. In addition to surgery, the most used first-line treatment for ovarian cancer is the chemotherapeutic agent cisplatin (CDDP) and paclitaxel. However, patients often develop chemoresistance after initial treatment, which reduces the efficiency of ovarian cancer treatment and increases its difficulty [3]. Therefore, it is necessary to develop natural active products that can either treat ovarian cancer or enhance the sensitivity to chemotherapeutic agents.

Natural products are highly valued in cancer drug research for their demonstrated anti-cancer effects, coupled with their abundant resources. Harmine (HM) is a tricyclic β-carboline alkaloid isolated from the seeds of *Peganum harmala* L. [4], a medicinal plant that produces a class of β-carboline alkaloids with diverse pharmacological activities, including anti-tumor, antioxidant, and anti-inflammatory properties [5]. Recently, HM has been shown to suppress ovarian cancer progression through various mechanisms, such as induction of autophagy/ferroptosis via the PI3K/AKT/mTOR pathway, inhibition of CDK9 to overcome PARP inhibitor resistance, and suppression of HDAC7 to block metastasis [6,7,8]. Harmine hydrochloride (HMH) is a plant-derived derivative of HM, which is more stable and can be absorbed more easily by tissues compared to HM [9]. Indeed, HMH has been reported to suppress liver and breast cancer via cell cycle arrest and mitochondrial apoptosis [10,11,12], and to inhibit gastric cancer by modulating proliferation, apoptosis, invasion, and migration [13]. However, whether HMH exerts any effect against ovarian cancer, and what its direct molecular target might be, remain entirely unexplored.

The nuclear factor of activated T cells (NFAT) family comprises five transcription factors (NFAT1–NFAT5) that are closely associated with T cell activation [14,15]. Among them, NFAT1 (NFATC2) was the first to be identified and has since been considered a promising target for immune response research [16]. However, beyond its classical role in immunity, NFAT1 has recently emerged as a critical oncogenic driver in multiple malignancies [17,18,19], including renal cancer [20], lung cancer [21], breast cancer [22,23], glioma [24,25], and colorectal cancer [26]. Indeed, NFAT1 drives tumor development through multiple mechanisms. For example, the NFAT1-MDM2 pathway was active in hepatocellular carcinoma (HCC) in humans [27]. The NFAT1/SOX2/ALDHA1 axis induces CSC phenotypes and drug resistance in lung adenocarcinoma [21]. Intriguingly, our preliminary analysis of clinical datasets indicated that elevated NFAT1 expression correlates with poor prognosis in ovarian cancer patients, yet its functional role in this disease has not been formally addressed. Hence, NFAT1 is seen as a highly attractive but yet-to-be-validated target for ovarian cancer intervention.

In the present study, we demonstrated that HMH can exert anti-ovarian cancer effects by inducing cell cycle arrest, promoting apoptosis, and suppressing cell migration in vitro. Importantly, HMH impeded intraperitoneal xenograft development, both as a monotherapy and in combination with cisplatin (CDDP). Additionally, we identified that NFAT1 promotes ovarian cancer progression and demonstrated its functional involvement in the anti-tumor activity of HMH. Collectively, these findings provide a strong rationale for developing HMH as a novel therapeutic candidate and highlight NFAT1 as a promising target for ovarian cancer treatment.

## 2. Results

### 2.1. HMH Suppresses Ovarian Cancer Cell Viability, Induces Apoptosis, Arrests Cell Cycle and Inhibits Migration In Vitro

**Figure 1:**
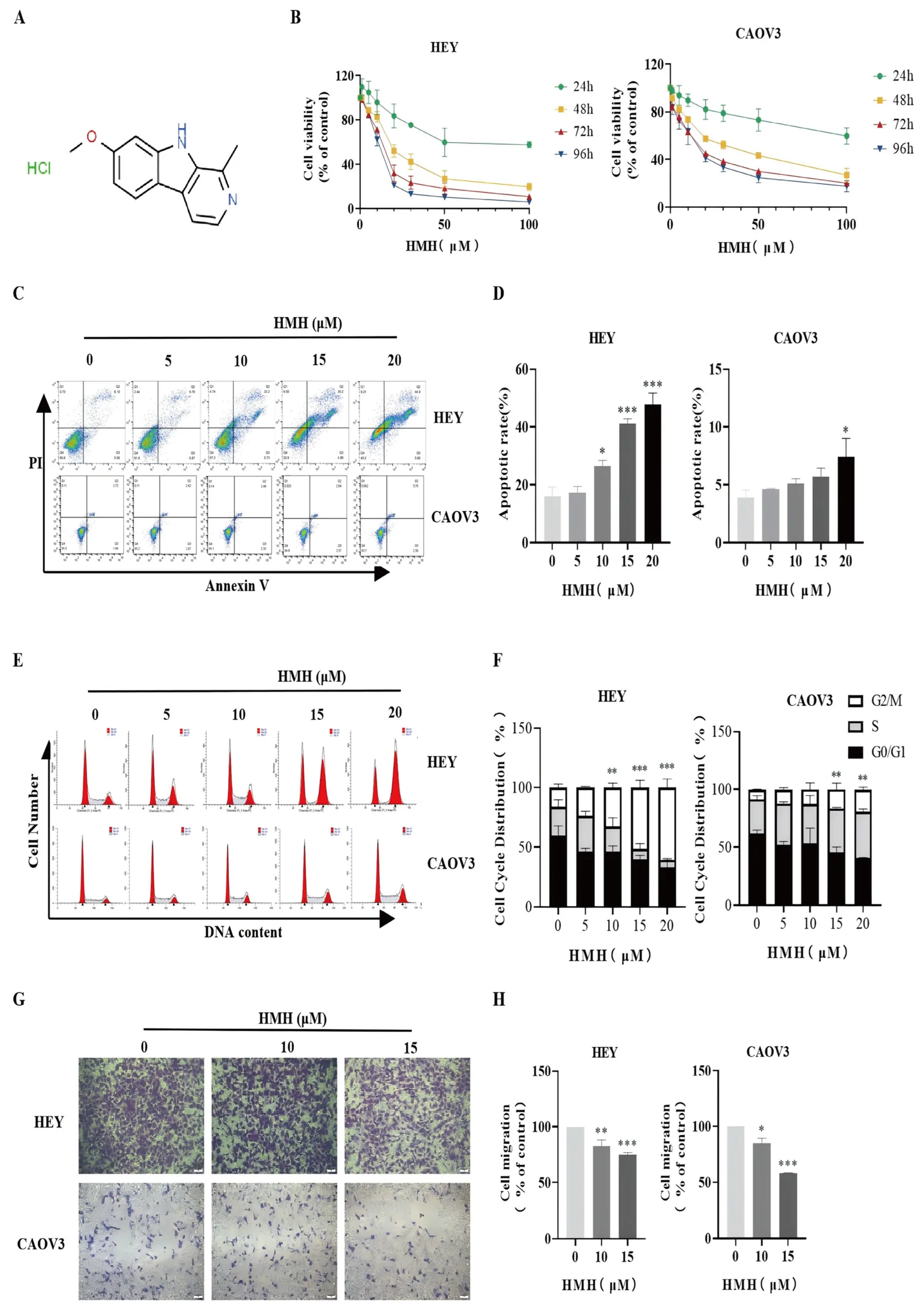
HMH inhibits ovarian cancer cell cycle, induces apoptosis and suppresses migration. (**A**) Chemical structure of HMH. (**B**) Cell viability rate of ovarian cancer cells at 24 h, 48 h, 72 h and 96 h with different concentrations (0, 1, 5, 10, 20, 30, 50 and 100 μM) of HMH by CCK8 assay. (**C**,**D**) HEY and CAOV3 cells were treated with HMH (0, 5, 10, 15 and 20 μM) for 48 h and the apoptosis was detected by flow cytometry. (**E**,**F**) HEY and CAOV3 cells were treated with HMH (0 μM, 5 μM, 10 μM, 15 μM and 20 μM) for 48 h and the cell cycle was detected by flow cytometry. (**G**,**H**) HEY and CAOV3 cells were treated with HMH (0 μM, 10 μM and 15 μM) for 24 h and the cell migration was detected by a cell migration assay (scale bars: 100 μm). Data are expressed as the mean ± SD from at least three independent experiments. * *p* < 0.05, ** *p* < 0.01, *** *p* < 0.001.

To examine the anti-ovarian cancer effects of HMH (chemical structure shown in Figure 1A), we initially assessed cell viability in human ovarian cancer cell lines HEY, CAOV3, ES2, and SKOV3 following HMH treatment (HEY was derived from a peritoneal metastasis of a moderately differentiated papillary cystadenocarcinoma; CAOV3 and SKOV3 are serous adenocarcinoma cell lines; and ES2 is a clear cell carcinoma cell line), as well as in non-malignant immortalized ovarian surface epithelial cells (IOSE80) following HMH treatment. As shown in Figure 1B and Figure S1, HMH suppressed cell growth in a time- and concentration-dependent manner. Compared with IOSE80, the cancer cell lines were generally more sensitive to HMH, indicating preferential activity against ovarian cancer cells. Then, to determine whether HMH inhibited cell growth via inducing apoptosis and blocking cell cycle in ovarian cancer cells, we treated HEY and CAOV3 cells with HMH. The results showed that 20 μM HMH caused cell apoptosis and significantly arrested the cell cycle at the G2/M phase in both HEY and CAOV3 cells (Figure 1C–F). In addition, a cell migration assay was used to evaluate cell migratory capacity after a 24 h HMH treatment. The results demonstrated that HMH could inhibit cell migration, with the migration rate of CAOV3 cells being reduced to only 57.79% at 15 μM (Figure 1G,H). Collectively, these results suggest that HMH can exert anti-ovarian cancer effects by inducing apoptosis, blocking cell cycle, and inhibiting cell migration.

### 2.2. RNA-Seq Shows That HMH Regulates Apoptosis and Cell Cycle in Ovarian Cancer Cells

**Figure 2:**
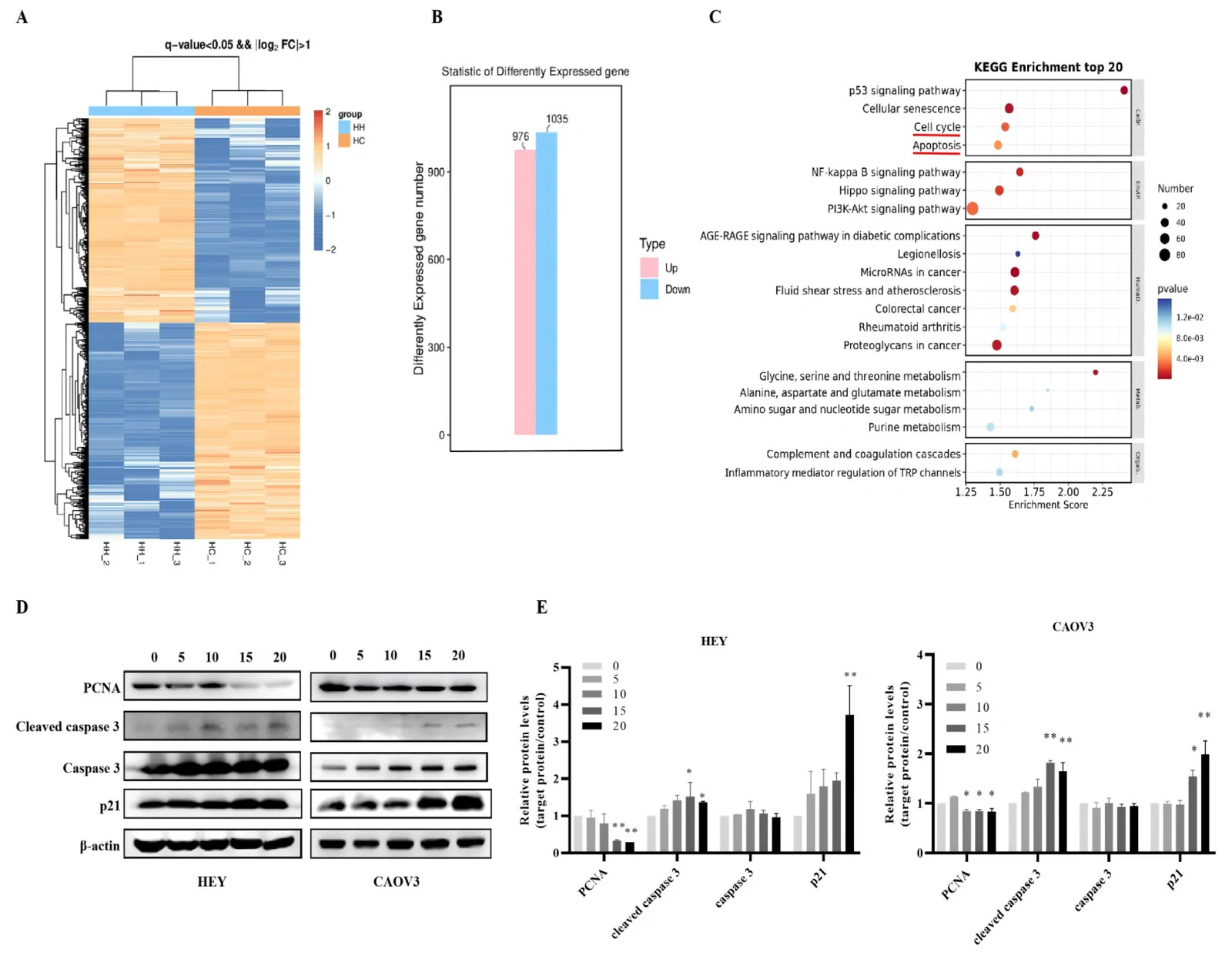
RNA-seq shows that HMH regulates apoptosis and cell cycle in ovarian cancer cells. (**A**) Results of cluster analysis of differential genes in the HH and HC. (**B**) Differential gene statistical map. (**C**) KEGG enrichment bubble diagram. (**D**,**E**) Cells were treated with HMH (0, 5, 10, 15 and 20 μM) for 48 h and analyzed by Western blot. Data are expressed as the mean ± SD from at least three independent experiments. * *p* < 0.05, ** *p* < 0.01.

To further elucidate the molecular mechanism underlying the anti-ovarian cancer effect of HMH, we conducted an RNA-seq analysis on HEY cells treated with 20 μM HMH for 48 h (HH), comparing them to the solvent control group (HC). Differential gene clustering analysis revealed that 976 genes were up-regulated and 1035 were down-regulated compared with the control group (Figure 2A,B). Kyoto Encyclopedia of Genes and Genomes (KEGG) pathway enrichment analysis indicated that the differentially expressed genes were predominantly involved in the apoptosis and cell cycle, with a significance threshold of *p* < 0.05 (Figure 2C). Western blot further confirmed that HMH significantly increased the expression associated with cell cycle and apoptotic related proteins such as p21 and cleaved caspase 3, while reducing the expression of PCNA (Figure 2D,E). These results are consistent with the above findings.

### 2.3. HMH Down-Regulated the Expression of NFAT1 in Ovarian Cancer Cells

**Figure 3:**
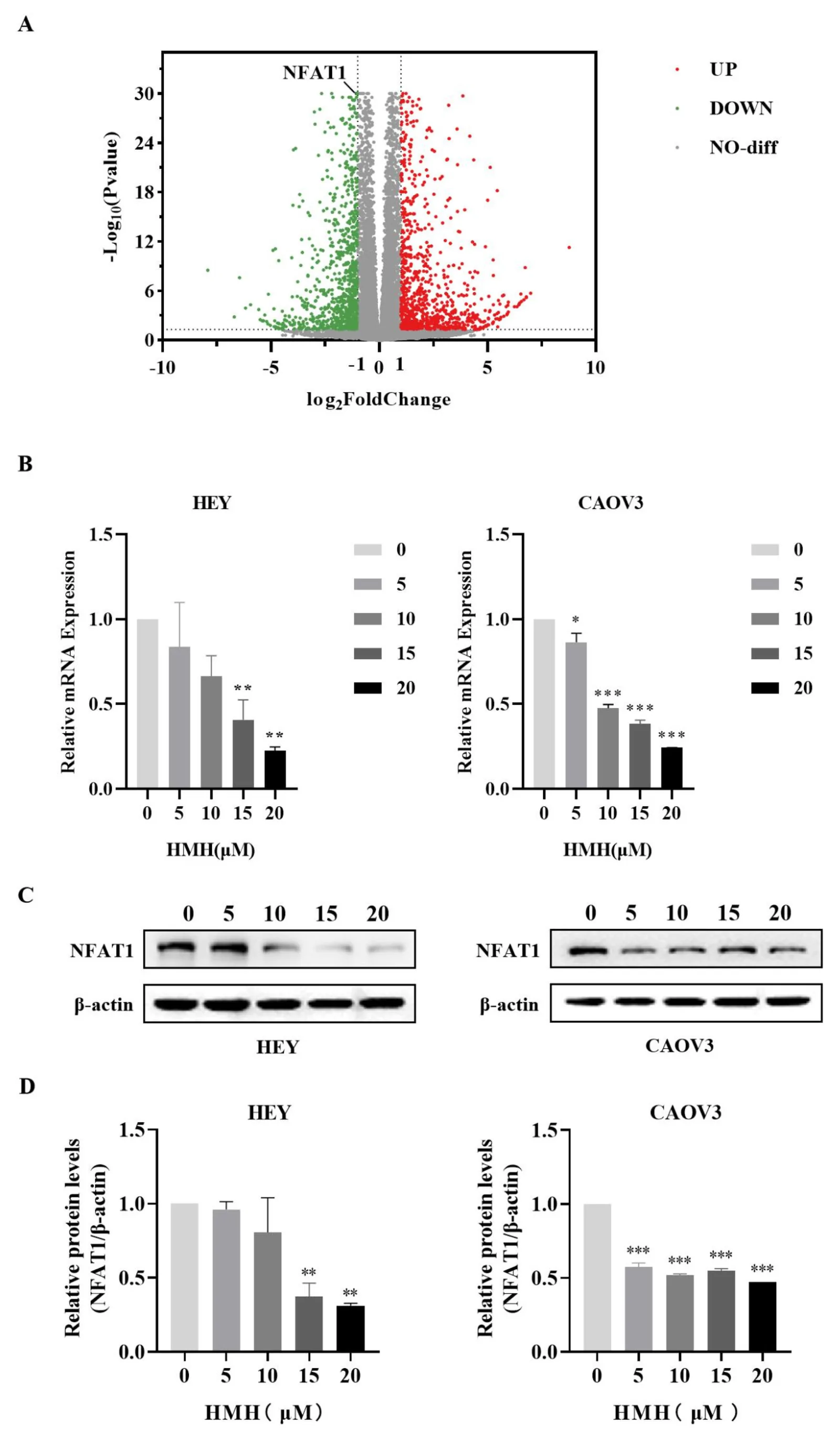
HMH suppresses NFAT1 expression at both the mRNA and protein levels. (**A**) Differential gene volcano map. (**B**) Cells were treated with HMH (0 μM, 5 μM, 10 μM, 15 μM and 20 μM) for 48 h and analyzed by qRT-PCR. (**C**,**D**) Cells were treated with HMH (0 μM, 5 μM, 10 μM, 15 μM and 20 μM) for 48 h and analyzed by Western blot. Data are expressed as the mean ± SD from at least three independent experiments. * *p* < 0.05, ** *p* < 0.01, *** *p* < 0.001.

Given the implication of calcineurin-NFAT signaling in harmine-induced beta cell proliferation [28] and our RNA-seq finding that 20 μM HMH significantly down-regulated NFAT1 gene expression in HEY cells (Figure 3A), we hypothesized that HMH exerts its anti-ovarian cancer effects through NFAT1. Subsequently, we employed qRT-PCR to verify the RNA-seq findings. Compared to the control group, HMH markedly reduced NFAT1 mRNA levels in HEY and CAOV3 cells in a dose-dependent manner (Figure 3B). Meanwhile, HMH also diminished NFAT1 protein levels in ovarian cancer cells (Figure 3C,D). These results suggest that downregulation of NFAT1 is at least one of the mechanisms by which HMH exerts its anti-ovarian cancer effects.

### 2.4. NFAT1 Promotes Ovarian Cancer Development and Is Clinically Associated with Patient Prognosis

**Figure 4:**
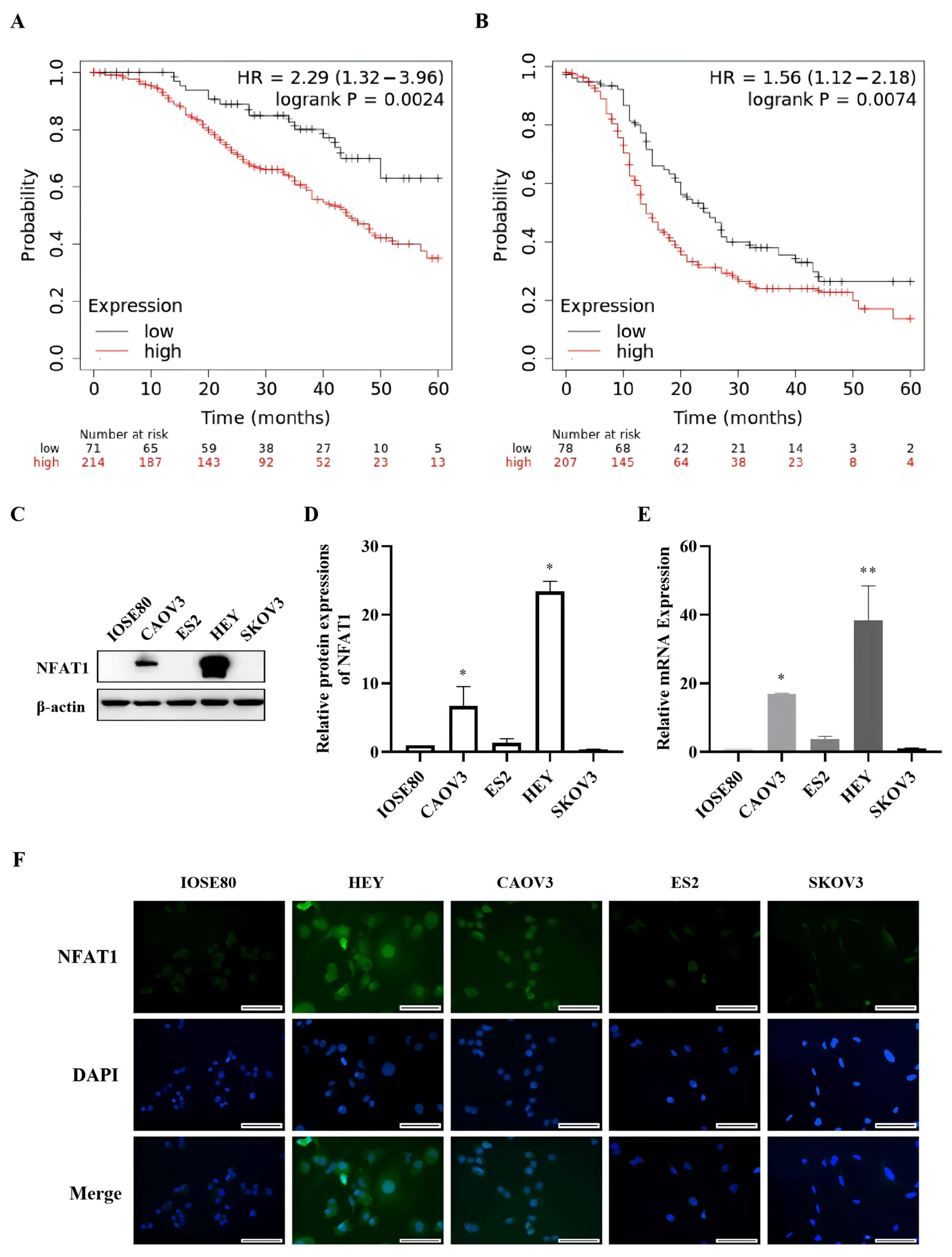
NFAT1 expression in ovarian cancers and is clinically associated with patient prognosis. Effect of NFAT1 on OS (**A**) and PFS (**B**) in ovarian cancer patients. Western blot (**C**,**D**) and qRT-PCR analysis (**E**) of NFAT1 expression in normal ovarian epithelial cells IOSE80 and ovarian cancer cells CAOV3, ES2, HEY and SKOV3. (**F**) The expression and distribution of NFAT1 was analyzed by IF (scale bars: 100 µm). Data are expressed as the mean ± SD from at least three independent experiments. * *p* < 0.05, ** *p* < 0.01.

Although NFAT1 has been closely associated with cancer development, its role in ovarian cancer has not been extensively studied. Kaplan–Meier curves were utilized to assess the relationship between NFAT1 expression and the prognosis of ovarian cancer patients. The findings indicated that patients with low NFAT1 expression levels had significantly improved overall survival (OS) and progression-free survival (PFS) (Figure 4A,B). To further investigate the relationship between NFAT1 and ovarian cancer, we assessed the expression and distribution of NFAT1 in normal ovarian epithelial cells (IOSE80) and ovarian cancer cells (HEY, CAOV3, ES2 and SKOV3) using qRT-PCR, Western blot and IF. NFAT1 was found to be highly expressed in HEY and CAOV3 at both protein and mRNA levels compared to IOSE80, while it exhibited low expression in SKOV3 and ES2 cells (Figure 4C–E). The IF results further showed that NFAT1 is primarily localized to the cell nucleus in both HEY and CAOV3 cells (Figure 4F). This finding is consistent with other literature reports, which state that NFAT1 is abnormally expressed in the nucleus of tumors [29].

**Figure 5:**
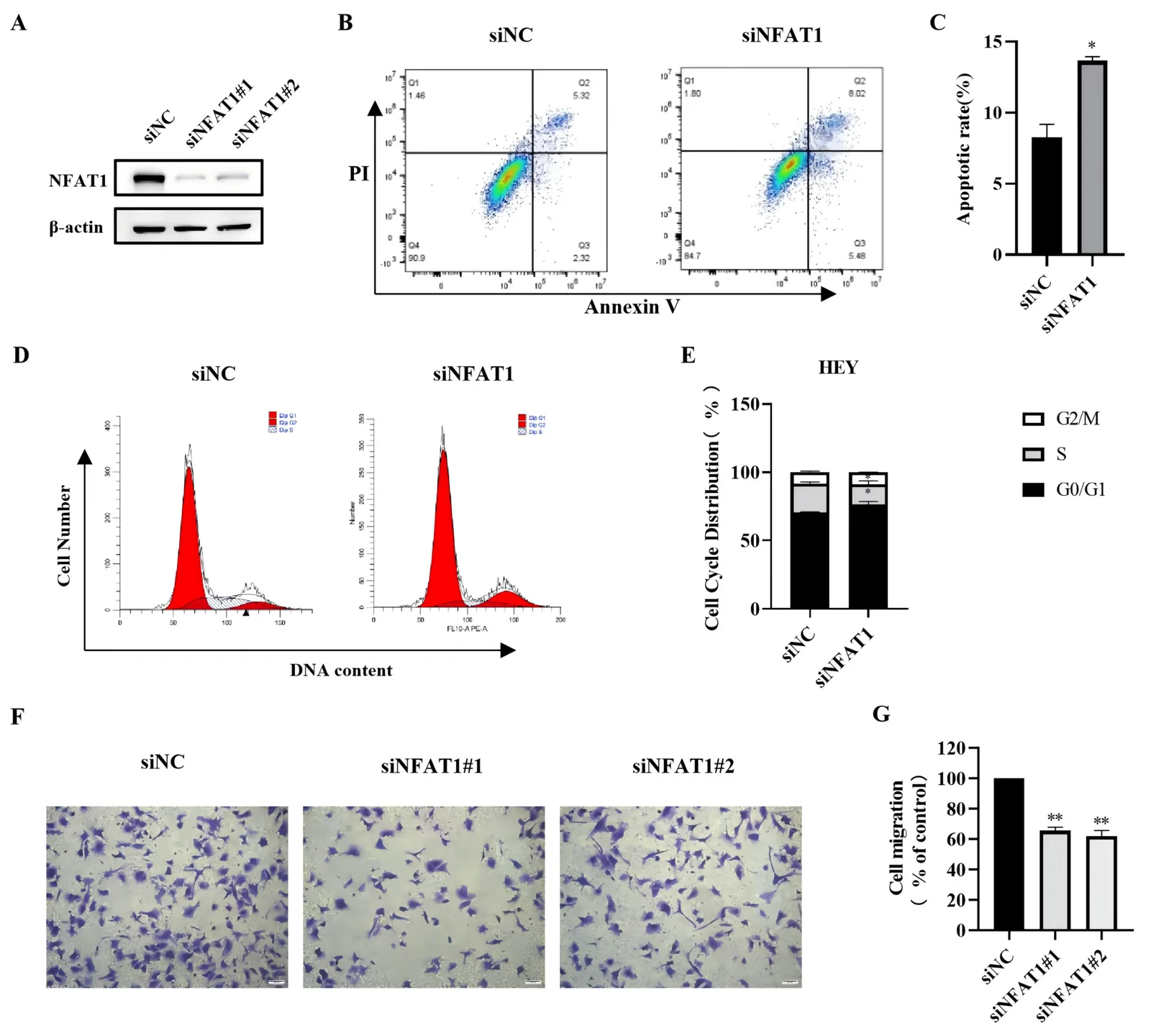
NFAT1’s impact on ovarian cancer apoptosis, cell cycle arrest, and migration. (**A**) Western blot of NFAT1 silencing in ovarian cancer cells after 48 h of siRNA. Effects on apoptosis (**B**,**C**) and cell cycle (**D**,**E**) were detected by flow cytometry after NFAT1 knockdown. (**F**,**G**) Migration effects of ovarian cancer cells after NFAT1 knockdown (scale bars: 100 µm). Data are expressed as the mean ± SD from at least three independent experiments. * *p* < 0.05, ** *p* < 0.01.

Based on the above outcomes, HEY and CAOV3, which exhibited high NFAT1 expression, were chosen for siRNA experiments. As depicted in Figure 5A and Figure S2A, the protein expression of NFAT1 was significantly reduced compared with the siNC in both HEY and CAOV3 cells. Subsequently, the impact of NFAT1 on apoptosis and cell cycle was evaluated using flow cytometry. Figure 5B,C illustrated that while the apoptosis rate in the siNC was 8.28% for HEY cells, this rate increased to 13.66% upon NFAT1 silencing. However, knockdown of NFAT1 did not affect the apoptosis in CAOV3 cells (Figure S2B,C). Furthermore, NFAT1 silencing led to a statistically significant increase in the G0/G1 phase ratio in both HEY and CAOV3 cells (Figure 5D,E and Figure S2D,E). Additionally, a cell migration assay demonstrated that NFAT1 knockdown repressed the migratory capacity of ovarian cancer cells (Figure 5F,G and Figure S2F,G). Collectively, these findings suggest NFAT1 promotes ovarian cancer development and is clinically associated with patient prognosis.

### 2.5. HMH Suppresses NFAT1 Nuclear Localization and Reverses Ion-Induced NFAT1 Nuclear Accumulation

**Figure 6:**
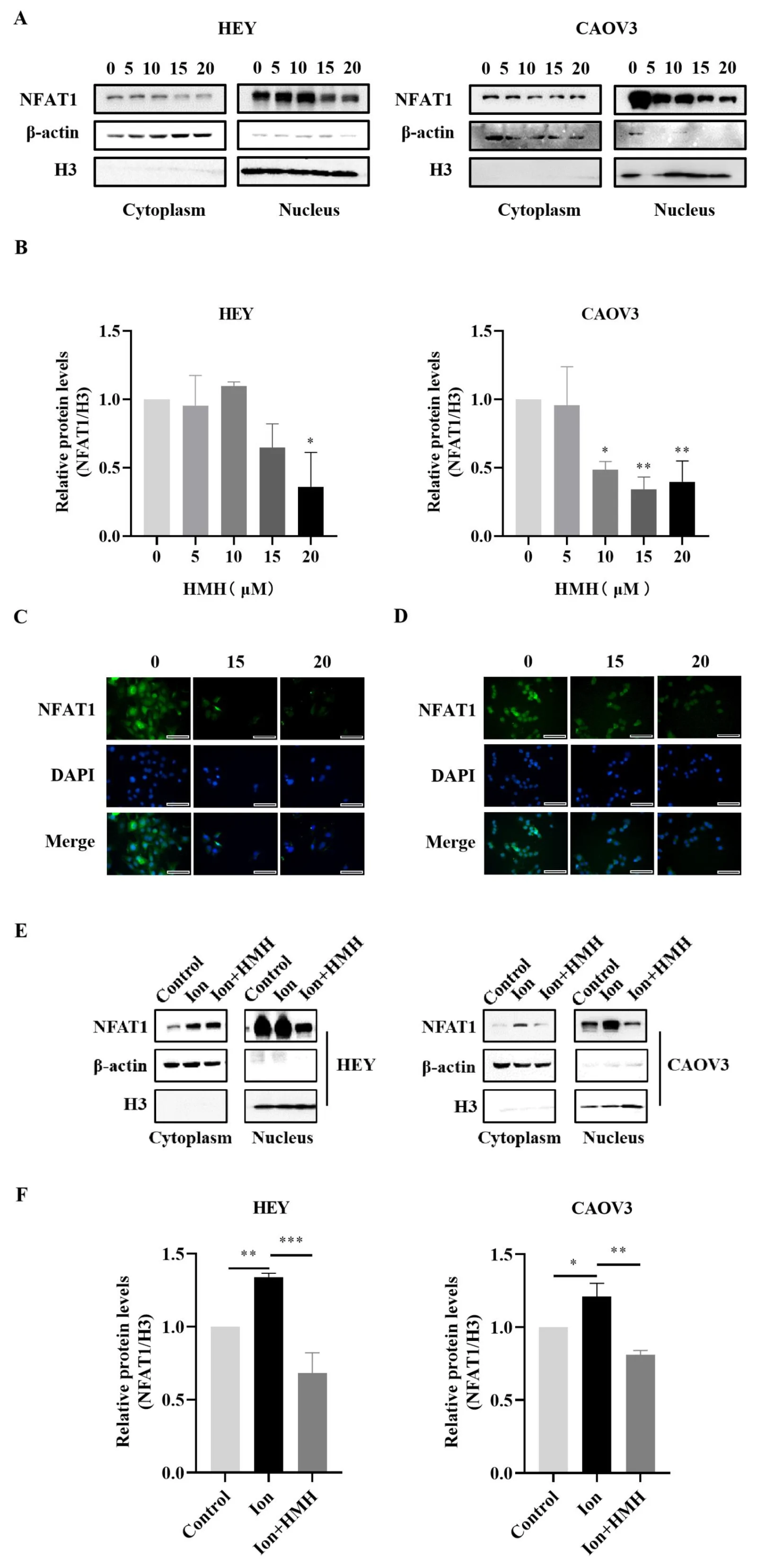
HMH reduces NFAT1 expression and reverses Ion-induced NFAT1 levels in ovarian cancer cell nuclei. (**A**,**B**) Cells were treated with HMH (0 μM, 5 μM, 10 μM, 15 μM and 20 μM) for 48 h, and the abundance of nuclear and cytoplasmic NFAT1 proteins was analyzed by Western blot. (**C**,**D**) Cells were treated with HMH (0 μM, 15 μM and 20 μM) for 48 h, and the abundance of nuclear and cytoplasmic NFAT1 proteins was analyzed by IF (scale bars: 100 µm). (**E**,**F**) Western blot was used to determine the effect of HMH on NFAT1 expression induced by Ion in ovarian cancer cells. Data are expressed as the mean ± SD from at least three independent experiments. * *p* < 0.05, ** *p* < 0.01, *** *p* < 0.001.

NFAT1 is a transcription factor. Based on this, we hypothesized that HMH might exert its anti-cancer effects by targeting the nuclear abundance of NFAT1. Western blot and IF results showed that NFAT1 was highly expressed in the nuclei of ovarian cancer cells in the control group, while HMH significantly suppressed NFAT1 protein expression within the nucleus (Figure 6A–D). NFAT1 can be activated in response to calcineurin (CaN), leading to NFAT1 dephosphorylation and translocation into the nucleus to exert transcriptional regulation [30]. Ion was able to promote the activation of NFAT1 and increase its abundance in the nucleus [29]. Importantly, HMH effectively reversed the Ion-induced increase in nuclear NFAT1 levels (Figure 6E,F). This indicates that NFAT1 is abnormally activated in ovarian cancer and NFAT1 plays a significant role in the anti-ovarian cancer effects of HMH.

### 2.6. HMH Binds to NFAT1 and Induces Its Proteasomal Degradation

**Figure 7:**
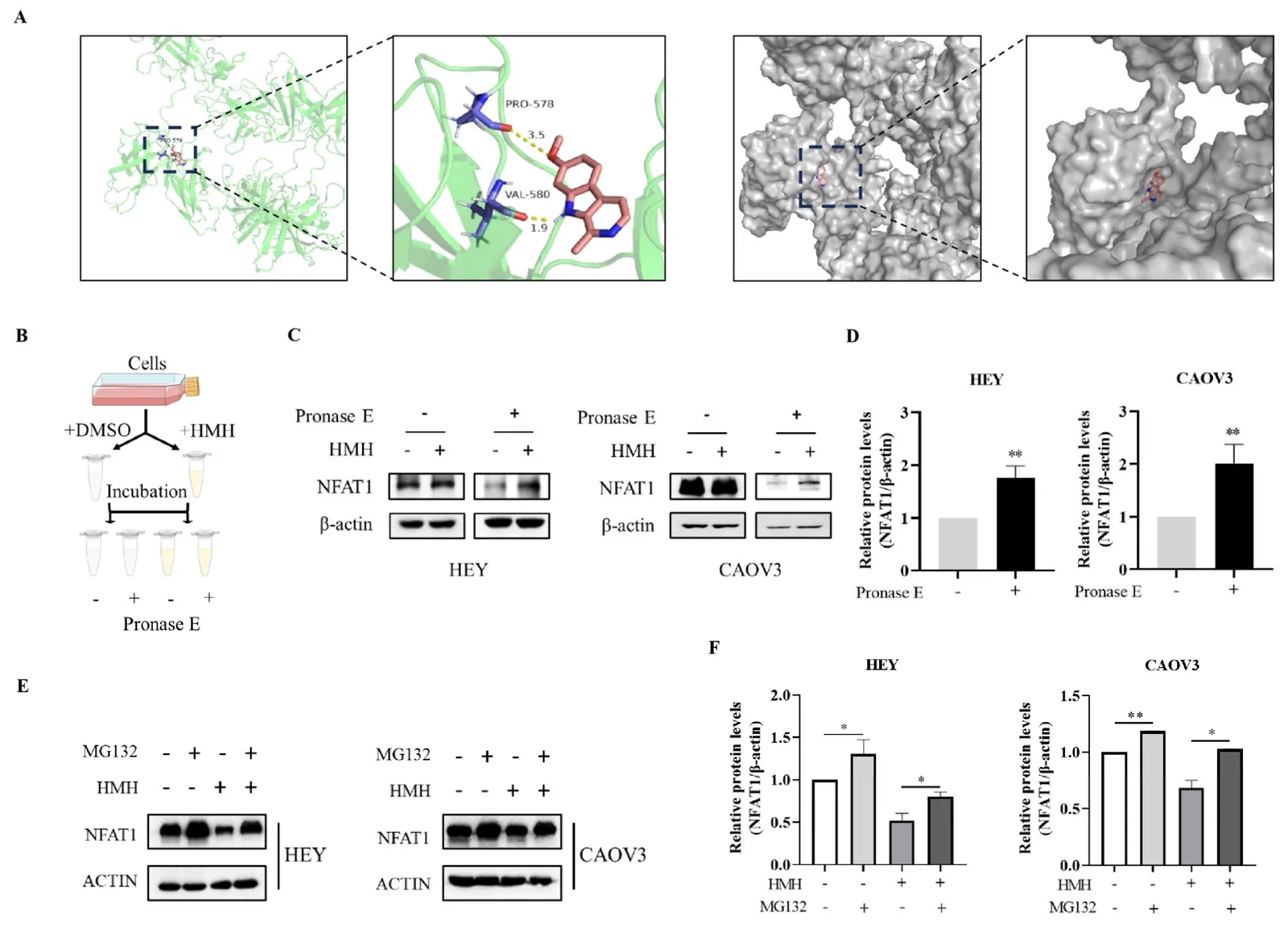
HMH binds to NFAT1 and induces its proteasomal degradation. (**A**) Molecular docking simulates the visualization of the binding site between HMH and NFAT1. (**B**) Flowchart of DARTS. (**C**,**D**) Western blot showed that HMH increases the stability of NFAT1 to the enzyme in ovarian cancer cells. (**E**,**F**) Ovarian cancer cells were treated with HMH (20 μM) or DMSO (vehicle) for 48 h, then exposed to MG132 (20 μM) for an additional 6 h. The protein levels of NFAT1 were detected by Western blot. Data are expressed as the mean ± SD from at least three independent experiments. * *p* < 0.05, ** *p* < 0.01.

We hypothesized that HMH could bind to NFAT1, a notion confirmed through molecular docking with AutoDock 1.5.7. The docking, visualized in PyMOL(v2.5), yielded a binding energy of −4.25 kcal/mol (1 kcal = 4.2 kJ), indicating a moderate and spontaneous binding potential between HMH and NFAT1 (Figure 7A). The figure depicts the amino acids at the binding sites of the small molecule ligand HMH and the receptor NFAT1 (Figure 7A). Notably, the backbone amide of VAL-580 forms a hydrogen bond with HMH, whereas PRO-578, whose backbone imino nitrogen cannot serve as a hydrogen-bond donor, contributes to ligand binding mainly through hydrophobic and van der Waals interactions. Together, these residues form a binding pocket within NFAT1 (Figure 7A). So, we proposed that HMH exerts anti-ovarian cancer effects via its binding to NFAT1, which was further validated using DARTS, as illustrated by the flowchart in Figure 7B. Western blot of DARTS samples revealed that HMH at 200 µM enhanced the resistance of NFAT1 to proteolytic degradation in ovarian cancer cells (Figure 7C,D). To determine whether the HMH-induced loss of NFAT1 protein occurs via the ubiquitin-proteasome pathway, we treated ovarian cancer cells with the proteasome inhibitor MG132 and examined NFAT1 protein levels. The outcomes showed that the NFAT1 reduction induced by HMH was prevented by MG-132 treatment (Figure 7E,F). These findings suggest that HMH probably promotes the proteasomal degradation of NFAT1 following direct binding.

### 2.7. HMH Suppresses Intraperitoneal Xenograft Growth and Reduces NFAT1 Expression In Vivo

**Figure 8:**
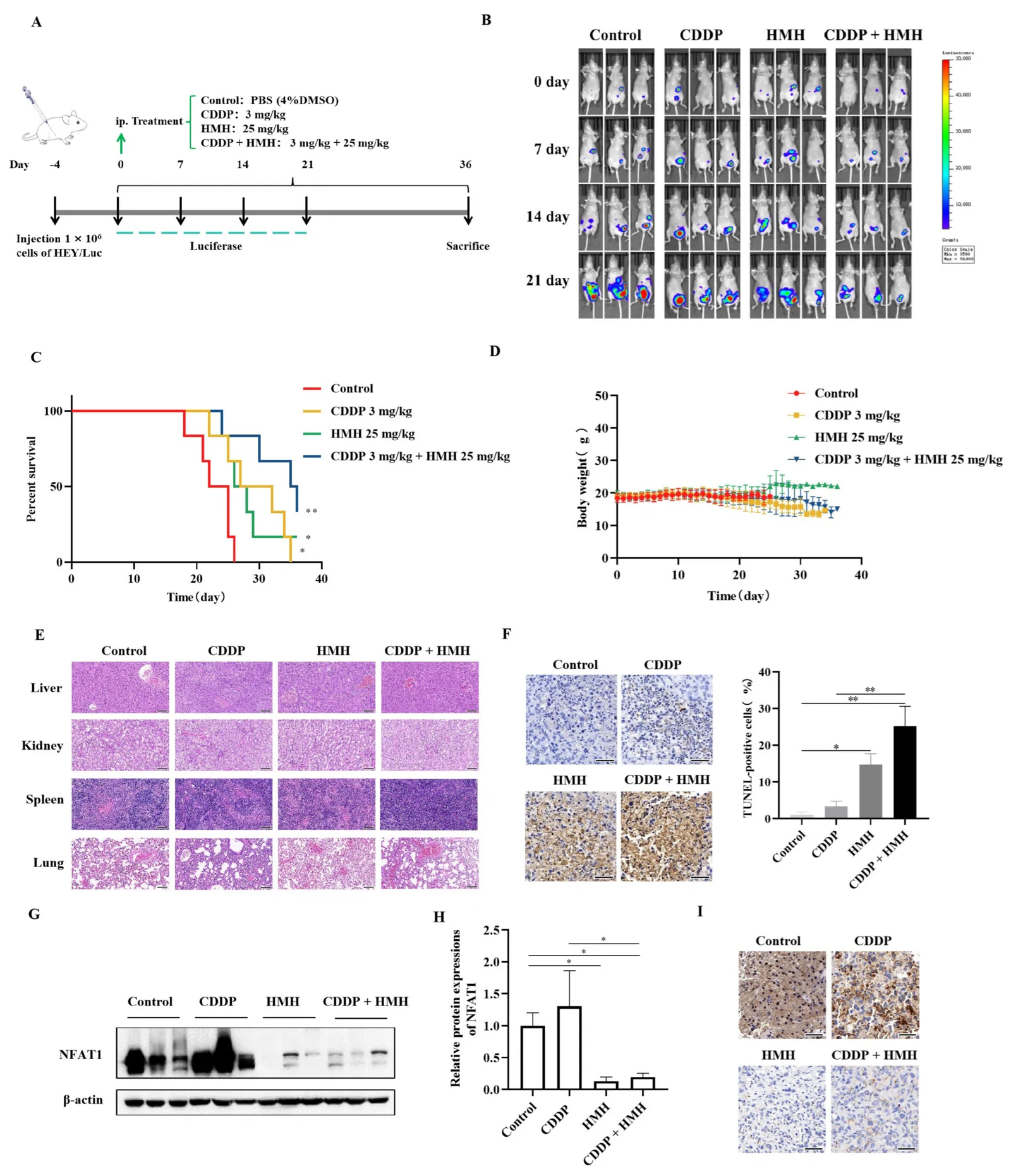
HMH inhibits intraperitoneal xenograft development. (**A**) Flowchart of therapy scheme (n = 6). (**B**) HEY/Luc cells (1 × 10^6^ cells/mouse) were injected intraperitoneally into nude mice for 4 days, followed by administration of HMH or CDDP. Tumors were imaged every week in the Xenogen system. (**C**) Kaplan–Meier analysis of animal endpoint survival following treatment with HMH and CDDP. (**D**) Body weight was monitored every day. (**E**) The liver, kidney, spleen and lung tissues were stained with H&E (scale bars: 100 µm). (**F**) The apoptosis of tumor cells was observed by TUNEL staining (scale bars: 50 µm). (**G**,**H**) NFAT1 protein expression in tumor tissues was assessed by Western blot. (**I**) Representative pictures of IHC staining of NFAT1 in tumor tissues (scale bars: 50 µm). Data are expressed as the mean ± SD from at least three independent experiments. * *p* < 0.05, ** *p* < 0.01.

To confirm our previous findings in vitro, we investigated the effect of HMH on intraperitoneal xenograft development of luciferase-expressing HEY (HEY/Luc) cells (the therapeutic scheme is depicted in Figure 8A). Bioluminescence imaging showed that intraperitoneal xenografts were detected 4 days after HEY/Luc cell injection (Figure 8B). Compared with the control group (vehicle treated), administering HMH (25 mg/kg) deterred tumor development and prolonged the survival of tumor-bearing mice (Figure 8B,C). Furthermore, the combination of HMH (25 mg/kg) and 3 mg/kg CDDP also deterred tumor development (Figure 8B). However, the combination therapy did not extend the survival of mice compared to the group treated with CDDP alone or HMH group alone (Figure 8C). Notably, while HMH alone did not affect body weight, its combination with CDDP markedly alleviated the CDDP-induced body weight reduction in tumor-bearing mice (Figure 8D). In addition, H&E staining showed that there was no significant toxicity in any vital organs including the liver, kidney, spleen, and lung after HMH administration (Figure 8E). To link HMH-led suppression in xenograft development to apoptosis, we analyzed TUNEL assay in the collected tumors. The results showed that HMH (25 mg/kg), either alone or in combination with CDDP, led to a significant increase in TUNEL-positive cells compared to the control group (Figure 8F). Western blot and IHC further revealed weaker NFAT1 expression in the HMH-treated groups compared to the control group, with similar results observed in the combination group (Figure 8G–I). These results suggest that HMH suppresses intraperitoneal xenograft development of ovarian cancer cells by inducing apoptosis and reducing NFAT1 expression.

## 3. Discussion

Natural products derived from plants have long been recognized as an important source of therapeutic drugs, particularly for anti-cancer agents [31,32]. In our research, for the first time, we found that HMH, derived from the seeds of *Peganum harmala* L., possesses an anti-ovarian cancer effect. First, HMH can arrest the cell cycle, induce apoptosis, and suppress cell migration in vitro. Second, NFAT1 promotes ovarian cancer progression. Third, HMH exerts its effects on NFAT1 through not only a reduction in its transcriptional level but also direct binding and the induction of its proteasomal degradation. Finally, we reveal that HMH impedes intraperitoneal xenograft development, either when used alone or in combination with CDDP. These results not only demonstrate the therapeutic potential of HMH as an agent against ovarian cancer, but also support the concept that targeting NFAT1 may represent a promising therapeutic strategy for ovarian cancer.

Numerous studies have elucidated the association between dysregulated NFAT1 signaling and diverse types of cancer cells [19,33,34]. Notably, other members of the NFAT family and the upstream calcineurin/NFAT signaling axis have been implicated in ovarian cancer. NFATc1 was reported to be overexpressed in ovarian cancer tissues and to promote cell growth and tumorigenesis by up-regulating c-Myc through the ERK1/2/p38 MAPK pathway [35], and NFATc1 depletion reduced the proliferation, migration, invasion and angiogenesis of SKOV3 ovarian cancer cells both in vitro and in vivo [36]. In addition, high expression of calcineurin, the phosphatase responsible for NFAT dephosphorylation and nuclear translocation, has been associated with poor prognosis in specific subtypes of ovarian cancer [37]. These studies collectively indicate that the calcineurin/NFAT pathway participates in ovarian cancer progression, while the specific role of NFAT1 in this disease has remained largely unexplored, which underscores the novelty of the present study.

Our data, presented through Kaplan–Meier curves, demonstrated that patients with low NFAT1 expression levels had significantly improved OS and PFS. This indicates that NFAT1 is clinically associated with patient prognosis and deserves further study in ovarian cancer. The Gene Expression Profiling Interactive Analysis (GEPIA) database analysis showed that NFAT1 was found highly expressed in a variety of cancers, such as ovarian, lung and esophageal cancers [33]. Fortunately, our experiments utilizing qRT-PCR and Western blot confirmed that the expression of NFAT1 in certain ovarian cancer cells was significantly higher than that in normal epithelial ovarian cells. Additionally, the IF and IHC assays demonstrated that NFAT1 is localized in both the nucleus and the cytoplasm, with a predominant nuclear localization. Increasing evidence reported NFAT1 promoted cancer cell growth, cell cycle progression, migration, invasion, and angiogenesis through calcineurin-dependent and -independent pathways across various cancer types. This includes its involvement in breast cancer, glioma stem-like cells, and melanoma, highlighting NFAT1’s important role in cancer progression [25,38,39]. In our experiments, we found similar results that knockdown of NFAT1 induced cell cycle arrest and triggered apoptosis and significantly decreased cell migration in ovarian cancer. Therefore, targeting NFAT1 may be a promising strategy for developing novel and effective anti-ovarian cancer therapy. Surprisingly, HMH arrests ovarian cancer cells in the G2/M phase, but the silencing of NFAT1 arrests ovarian cancer cells in the G0/G1 phase. This implies that HMH potentially induces cell cycle arrest via alternative pathways, necessitating additional investigation.

In our study, a series of experiments demonstrated that HMH could inhibit NFAT1 expression in the nucleus of HEY and CAOV3 cells. In the cell viability assay, we were surprised to find that HMH showed greater sensitivity towards cells with high NFAT1 expression, such as HEY and CAOV3, compared to those with low NFAT1 expression. These findings are more in line with our hypothesis that NFAT1 might be associated with the anti-ovarian cancer effect of HMH. Molecular docking and DARTS results further indicated that NFAT1 may be a direct target of HMH, while MG132 treatment blocked the HMH-induced loss of NFAT1 protein, indicating that proteasomal degradation contributes to the reduction in NFAT1 at the protein level.

Nevertheless, several limitations of this study should be acknowledged. First, although the molecular docking, DARTS and MG132 experiments consistently support a direct interaction between HMH and NFAT1, these approaches provide supportive rather than conclusive evidence of direct target engagement. Biophysical assays such as surface plasmon resonance (SPR) or the cellular thermal shift assay (CETSA) are warranted to determine the binding affinity and to validate target engagement in cells. Second, rescue experiments, such as re-expression or overexpression of NFAT1 in HMH-treated cells, were not performed in the present study. Such experiments would provide stronger evidence that the observed biological effects of HMH are primarily mediated through NFAT1. Moreover, how HMH induces the transcriptional downregulation of NFAT—whether by affecting upstream kinases or directly modulating the promoter activity of the NFAT1 gene—remains a crucial question for future investigation. This also exemplifies the multi-level and multi-pathway characteristics of natural products in exerting their anti-tumor effects.

Although CDDP is the most commonly used first-line treatment for ovarian cancer, the likelihood of developing drug resistance increases with ongoing treatment, and it can cause side effects [40]. Our in vivo data showed that HMH not only exerts effects against ovarian cancer on its own, but also demonstrates better efficacy when used in combination with CDDP (3 mg/kg), yielding superior results compared to the use of HMH or CDDP alone. Increasing evidence has revealed the efficacy of natural products in combination with other therapeutic agents [32], which provides new insights into novel therapeutic strategies in cancer treatment.

## 4. Materials and Methods

### 4.1. Cell Cultures and Reagents

Human HEY, CAOV3, ES2, SKOV3, and IOSE80 cells were purchased from Procell Life Science & Technology Co., Ltd. (Wuhan, China). HEY and CAOV3 cells were grown in DMEM (DMEM, meilunbio, Dalian, Liaoning, China). ES2 and SKOV3 cells were grown in McCoy’5A (meilunbio, Shanghai, China). IOSE80 cells were grown in RPMI-1640 (meilunbio, China). All media were supplemented with 1% non-essential amino acids (Beyotime, Shanghai, China) and 10% fetal bovine serum (FBS, BDBIO, Hangzhou, China) in an incubator containing 5% CO_2_ at 37 °C. HMH was purchased from Shanghai Tongtian Biotechnology Co., Ltd. (Shanghai, China). MG132 (HY-13259) and Ion (HY-13434) were purchased from MCE (MCE, MedChemExpress, Monmouth Junction, NJ, USA).

### 4.2. Cell Viability Assay

HEY, CAOV3, ES2, SKOV3 and ISOE80 cells were seeded in 96-well plates (3000 cells in 100 μL) and, after overnight adherence, exposed to HMH (0, 1, 5, 10, 20, 30, 50 and 100 μM) for 24 h, 48 h, 72 h and 96 h. Each concentration was tested in triplicate wells, and blank wells containing medium and CCK-8 reagent without cells were included for background correction. Cell viability was determined using the cell counting kit-8 (CCK-8, DOJINDO, Kumamoto, Japan). Following treatment, 10 μL of CCK-8 reagent was added to each well and incubated for 2 h at 37 °C, and the absorbance was then measured at 450 nm using a microplate reader (Bio-Rad, Hercules, CA, USA).

### 4.3. Cell Apoptosis Assay and Cell Cycle Assay

HEY and CAOV3 cells were seeded in 6-well plates at a density of 1 × 10^5^ cells/mL and exposed to the HMH (0 μM, 5 μM, 10 μM, 15 μM and 20 μM) or transfected with siRNA for 48 h. Collecting cell culture solution and cells were washed and resuspended. Cells were stained with Annexin V-FITC and propidium iodide (PI) (MULTI SCIENCES, Hangzhou, China), and cell apoptosis was detected with flow cytometry (BECKMAN, Brea, CA, USA). Cells were stained with a cell cycle staining kit (MULTI SCIENCES, China), and the cell cycle was detected with flow cytometry (BECKMAN, USA).

### 4.4. Cell Migration Assay

A 24-well transwell plate with 8.0-μm pore membranes was utilized to examine the cell migration, as previously described [41]. After serum starvation for 12 h, cells were plated in the upper transwell chamber and treated with HMH (0, 10, and 15 μM) for 24 h. Cotton swabs were used to remove any remaining cells in the top chamber, after which the cells were fixed for 15 min with 4% paraformaldehyde and stained with crystal violet solution.

### 4.5. RNA Sequencing Analysis (RNA-Seq)

HEY cells were divided into two experimental groups: an untreated group (HC) and a 20 μM HMH-treated group (HH), both for 48 h. RNA was extracted from HEY cells using Trizol reagent (ThermoFisher, Waltham, MA, USA). The RNA samples were sent to OE Biotech Co., Ltd. (Shanghai, China) for further analysis. Three biological replicates were analyzed per group. Differential expression analysis was performed using DESeq2, with significance thresholds set at Q < 0.05 and |fold change| > 2. Hierarchical clustering and enrichment analyses (GO, KEGG, Reactome, and WikiPathways) were conducted using R (v3.2.0).

### 4.6. Quantitative Real-Time Polymerase Chain Reaction (qRT-PCR)

The qRT-PCR protocol was based on previous literature [41]. Briefly, RNA was extracted for purity analysis and quantification, followed by reverse transcription into cDNA. Primers, cDNA and SYBR Green were mixed into an amplification system and qPCR was performed. Relative expression was calculated using the formula 2^−ΔΔct^. Primer sequences are provided in Supplemental Table S1.

### 4.7. Western Blot

Western blot was performed as previously described [41]. Briefly, cells or tissues were effectively lysed by adding RIPA lysis buffer and centrifuged at 4 °C at 12,000× *g* for 10 min. The cell lysate was collected for quantification. Western blot was performed to detect the expression of NFAT1, histone H3 (H3) and β-actin using an ECL system (Tanon, 4200SF, Shanghai, China). Antibody information is provided in Supplemental Table S2.

### 4.8. Separation and Extraction of Cytoplasmic and Nuclear Proteins

Separating and extracting of cytoplasmic and nuclear proteins following drug treatment were carried out in accordance with the kit’s (Thermo Fisher Scientific, Waltham, MA, USA) instructions.

### 4.9. Immunofluorescence (IF)

HEY and CAOV3 cells were seeded in 96-well plates at a density of 3 × 10^3^ cells per well. After treatment with HMH (0, 15, and 20 μM) for 48 h, cells were fixed with paraformaldehyde and then permeabilized with Triton X-100. Subsequently, cells were blocked with 5% bovine serum albumin (BSA) for 1 h at room temperature, followed by incubation with primary antibody (1:50) overnight at 4 °C. After washing, cells were incubated with fluorescent-labeled secondary antibody (1:500) (Beyotime, Shanghai, China) for 1 h at room temperature. Finally, nuclei were counterstained with DAPI-containing mounting solution. Expression of NFAT1 fluorescence was detected using a Cytation 5 (BIOTEK, Winooski, VT, USA).

### 4.10. Transfection of Small Interfering RNAs

Non-specific control (NC) siRNA and small interfering RNAs (siNFAT1) targeting NFAT1 were purchased from GenePharma (Shanghai, China). A 20 μM stock solutions was prepared. Cells were seeded into 6-well plates at 1 × 10^5^ cells per well and allowed to reach approximately 50% confluence. Subsequently, the cells were transfected with siRNA using Lipofectamine™ 3000 (Thermo Fisher Scientific, Waltham, MA, USA), according to the manufacturer’s guidelines. The transfection mixture was removed after an 8 h incubation in antibiotic-free medium, and the cells were then incubated in fresh medium for an additional 48 h. The sequences are provided in Supplemental Table S3.

### 4.11. Auto-Dock Molecular Docking Analysis

The structure of HMH was downloaded from the PubChem database. Hydrogenation and optimization of molecule structure were performed using AutoDock Tools 1.5.7 software, and its format was exported as a ligand. The target NFAT1 (ID:1P7H) 3D structure was obtained from the PDB (http://www.rcsb.org/ accessed on 24 January 2024) database. This structure was chosen because it is the only high-resolution crystal structure of NFAT1 currently available in the public database, and it contains a well-defined surface pocket adjacent to the DNA-binding interface. Then, AutoDock Tools 1.5.7 software was utilized to perform hydrogenation and other operations, resulting in the receptor’s format being exported. Receptor and ligand were imported into AutoDock Tools 1.5.7 software to obtain the corresponding pockets for molecular docking, to obtain the affinity values of the parameter for evaluation of their binding situation, and to visualize the docking results by the open-source software PyMOL.

### 4.12. Drug Affinity Responsive Target Stability (DARTS)

The DARTS assay was developed based on a previous study [42]. Briefly, ovarian cancer cell lysates were collected for quantification. After the cell lysates were incubated with HMH or an equal amount of DMSO for 1 h at 37 °C, Pronase E was added to the lysates at a ratio of 1:1000 for half an hour at room temperature. The samples were used for Western blot.

### 4.13. Intraperitoneal Xenograft Development Assay

Six-week-old female nude mice were purchased from Shanghai Sippe-Bk Lab Animal Co., Ltd. (Shanghai, China). Intraperitoneal xenograft development was assessed as previously described [43]. Briefly, mice were injected intraperitoneally with 1 × 10^6^ luciferase-expressing HEY (HEY/Luc) cells per mouse, with 6 mice per group. After 4 days of live imaging to assess the success of the modeling, animals were randomly sorted into groups based on size and fluorescence intensity. Four groups of nude mice were established: a control group treated with PBS (4%DMSO), a CDDP group treated with 3 mg/kg CDDP, an HMH group treated with 25 mg/kg HMH, and a CDDP + HMH group treated with both 3 mg/kg CDDP and 25 mg/kg HMH. The administration methods included intraperitoneal injection (ip.) twice weekly for CDDP, and six weekly injections for both PBS and HMH. Tumor progression was monitored by examining fluorescence in a Xenogen IVIS-200 In Vivo Imaging System on a weekly basis. Visible implants in peritoneal cavities were harvested and fixed for further study.

### 4.14. Histological Analysis

Tumor tissues were paraffin-embedded and sectioned to a thickness of 5 μm. The sections were deparaffinized and hydrated before being subjected to antigen retrieval in a heat-mediated citrate buffer (Sangon Biotech, Shanghai, China). For immunohistochemical (IHC) staining, NFAT1 was determined with a primary antibody against NFAT1 and then detected using goat anti-rabbit secondary antibody following manufacturer’s instructions (Abcam, Waltham, MA, USA). The methods for terminal deoxynucleotidyl transferase dUTP nick end labeling (TUNEL) staining were carried out in accordance with the kit’s (Roche, South San Francisco, CA, USA) steps. The liver, kidney, spleen, and lungs of the mice were harvested and fixed with 4% paraformaldehyde. The fixed tissue samples were then embedded, sectioned, and stained with hematoxylin and eosin (H&E) staining.

### 4.15. Kaplan–Meier Survival Analysis

The prognostic value of NFAT1 in ovarian cancer was evaluated using the Kaplan–Meier Plotter database (https://kmplot.com/analysis/ accessed on 1 February 2024), which integrates gene expression and clinical follow-up data from GEO, EGA, and TCGA, including the GSE9891 dataset of 285 ovarian tumor samples. Patients were divided into high- and low-expression groups by the median NFAT1 level. OS and PFS were assessed by Kaplan–Meier curves with log-rank tests, and HRs with 95% CIs were calculated.

### 4.16. Statistical Analysis

Statistical analysis was performed using GraphPad Prism software (version 8.0). All data were expressed as mean ± SD from at least three independent experiments. Student’s *t*-test was used for comparison between two groups. One-way ANOVA was used for comparisons among more than two groups. Values of *p* < 0.05 were considered significant.

## 5. Conclusions

In conclusion, this study provides evidence that HMH possesses anti-ovarian cancer activity, which is mediated, at least in part, through targeting NFAT1 for proteasomal degradation, leading to cell-cycle arrest, apoptosis and suppression of migration in vitro, as well as inhibition of intraperitoneal xenograft growth and prolonged survival in vivo, both as a monotherapy and in combination with CDDP. Beyond characterizing HMH as a candidate NFAT1 inhibitor, our findings highlight NFAT1 as a potential prognostic biomarker and therapeutic target in ovarian cancer. Given the limitations discussed above, future studies should include biophysical validation of the HMH-NFAT1 interaction and NFAT1 rescue experiments, which together will facilitate the development of HMH as a novel therapeutic candidate for ovarian cancer.

## Data Availability

The original contributions presented in this study are included in the article/Supplementary Material. Further inquiries can be directed to the corresponding authors.

## Supplementary Materials

The following supporting information can be downloaded at: https://www.mdpi.com/article/10.3390/ph19091341/s1, Figure S1: HMH inhibits cells growth; Figure S2: NFAT1’s impact on ovarian cancer apoptosis, cell cycle arrest, and migration; Table S1: Primer sequence list for qRT-PCR; Table S2: Antibody information; Table S3: siRNA sequence list.
