# Supplementary material for: Harmine Hydrochloride from Peganum harmala L. Targets NFAT1 for Proteasomal Degradation to Suppress Ovarian Cancer Progression
Source: Pharmaceuticals (Basel). 2026 Aug 24;19(9):1341. doi: 10.3390/ph19091341 (PMC13610283; doi:10.3390/ph19091341)
Supplement: Supplementary file 1 [file pharmaceuticals-19-01341-s001.zip › Fig S figure legends.pdf]

Figure S1. HMH inhibits cells growth. Cell viability rate of cells (ISOE80,ES2 and SKOV3) at 24 h, 48 h, 72 h and 96 h with different concentrations (0, 1, 5, 10 , 20 , 30, 50 and 100 μM) of HMH by CCK8 assay. Data are expressed from the mean ± SD at least three independent experiments.

Figure S2. NFAT1's impact on ovarian cancer apoptosis, cell cycle arrest, and migration. (A) Western blot analysis of NFAT1 silencing in CAOV3 cell after 48 h of siRNA. Effects on apoptosis (B-C) and cell cycle (D-E) after NFAT1 knockdown in CAOV3 cell. (F-G) Migration effects of NFAT1 silencing in CAOV3 cell. Data are expressed as the mean ± SD from least three independent experiments. *$P < 0.05$, **$P < 0.01$.
