# Supplementary material for: Harmine Hydrochloride from Peganum harmala L. Targets NFAT1 for Proteasomal Degradation to Suppress Ovarian Cancer Progression
Source: Pharmaceuticals (Basel). 2026 Aug 24;19(9):1341. doi: 10.3390/ph19091341 (PMC13610283; doi:10.3390/ph19091341)
Supplement: Supplementary file 1 [file pharmaceuticals-19-01341-s001.zip › Supplementary tables.pdf]

Table S1

Primer sequence list for qRT-PCR

Gene	Primer sequence list (5'-3')
NFAT1-forward	AACAGCCTCGCCACGGACTC
NFAT1-reverse	TCGTAAGAGCCTGACTGACTGGAC
β -actin- forward	CCAGCTCACCATGGATGATG
β -actin- reverse	ATGCCGGAGCCGTTGTC

Table S2

Antibody information

Antibody	Information
NFAT1	Cell Signaling Technology, #5861
H3	Cell Signaling Technology, #4499
Cleaved Caspase-3	Cell Signaling Technology, #9664
Caspase-3	Cell Signaling Technology, #9665
p21	Cell Signaling Technology, #2947
β -actin	Bioworld technology, AP0060
PCNA	Cell Signaling Technology, #85118
HRP-conjugated Affinipure Goat Anti-MOUSE IgG (H+L)	Proteintech, SA00001-1
HRP-conjugated Affinipure Goat Anti-Rabbit IgG (H+L)	Proteintech, SA00001-2

Table S3

siRNA sequence list

siRNA	siRNA sequence list（5’-3’）
siNFAT1, #1-S	GCCCAUGGUUGAAAGACAATT
siNFAT1, #1-AS	UUGUCUUUCAACCAUGGGCTT
siNFAT1, #2-S	GUCCAAAGUUGUGUUUACUTT
siNFAT1, #2- AS	AGUAAACACAACUUUGGACTT
siNC, S	UUCUCCGAACGUGUCACGUTT
siNC, AS	ACGUGACACGUUCGGAGAATT
